# Diseases of Children

**Published:** 1891-10

**Authors:** E. Van Goidtsnoven

**Affiliations:** Atlanta, Ga.


### DISEASES OF CHILDREN.

(*Continued.*)

BY DR. E. VAN GOIDTSNOVEN, ATLANTA, GA.

### CHOLERA INFANTUM.

Cholera Infantum is recorded nowhere but in the United States. Search the medical literature of France, England and Germany, and you will not find it. May we not infer that cholera infantum, like the Asiatic cholera, has for its origin a local cause; a germ claiming American birth and citizenship; an hydrophilous comma whose favorite habitat, judging from statistical records, seem to be Philadelphia and her surroundings; a Yankee microbe wafting in the atmosphere, seeking for or awaiting a congenial morbific fermentation or pabulum to develop itself therein, and then pursue its nefarious work?

Cholera infantum is a specific disease of the nervous system affecting infants and very young children, characterized by vomiting, purging, *watery* evacuations, prostration, collapse. The common idea is that it is an evacuation to be arrested by astringents and opiates. Mothers as well as many practitioners are only too willing to hold the teeth responsible for any disease which may arise during their evolution, and most particularly for cholera infantum.

Another common error is to hold that children are more liable to cholera infantum in the second year than in the first. But this does not seem to be confirmed by statistical information. They lose sight of the fact that teething is a physiological process and not a disease. Morever every physician knows or ought to know that cholera infantum is met with in children, who have no teeth, who are not teething at the inception of the disease, who have all their deciduous teeth, except the last four molars ; or when showing no swelling, tenderness or irritation of the gums.

From personal experience I have come to the conclusion that the lesion is primarily one of the sympathetic system of nerves which govern all the processes of vegetative life, typefying a nervous exhaustion which presents many points of analogy with that attending Asiatic cholera. There is much evidence pointing to the conclusion that there are in cholera infantum, as in Asiatic cholera, spasms of the arterioles often so great as to cut off secretions from kidneys, with labored action of the heart, etc., and much venous congestion ; so that there is great effusion from the portal circulation; hence *watery evacuations.* It can be said that now the hydrocephaloid stage is reached. The vomiting and purging are so severe as to cause the child in a few hours to lose all resemblance to its former self. The stomach is so irritable as to eject everything taken into it, even a mouthful of cold water ; the thirst is great, the features become shrunken, and present that deathlike expression familiarly known as *facies hypocratica ;* the skin is cold and clammy ; the eyes are half closed ; the extremities, nose, chin, tongue, and ears become cold ; there is partial insensibility and twitchings and startings. Insensibility may continue until it amounts to coma and death. To the least attentive observer cholera infantum has a physiognomical presentation *sui generis* which, once seen, can never be forgotten.

Physiognomical presentations instruct the physician better than all the verbal information he can receive from the sick or his attendants ; and this is of incalculable value when one is ministering to a child. In all organized beings there is a natural or normal susceptibility called by some a normal irritability peculiar to the nervous system. This susceptibility is increased by nervous debility which renders the whole organism more subject to irritating causes. This is seen in a child with his health impaired by first, improper alimentation ; secondly, excessive heat ; thirdly, atmospheric vitiation ; fourthly, dentition. Cholera infantum is the outcome of any or all of these morbific conditions. Through the latter, the soil is prepared, the pabulum is formed and the germ thrives. Necropsies reveal the fact that the encephalon, in nearly all cases, is in a state of hyperæmia, and, in long protracted cases serous effusions have been met with in the ventricles and at the surface of that viscus.

As cholera infantum may thus be presented by any of the above named conditions, their respective treatment is laid down in my first paper on the diseases of children and the formulæ Nos. 3, 4 and 5 will in a great measure confront most of the emergencies. Vomiting contra indicates food of any kind and imperatively demands rest for the stomach and the intestines. Hence total abstinence for several hours until ejection and purging cease. In collapse, the following will be found of great service:

6. ℞ Siberian musk, gr. i.

Sulphate of Atropia, gr. 1-2 [illegible]0

Emulsion of Acacea, f. ʒ i.

M. S. As a dose every hour until reaction takes place. To allay pain and give rest to the intestinal tract, I often prescribe:

7. R. Chloranodyne (P. D. & Co. gtt. i.
Aquæ f. ʒ i.

M. S. As a dose every four (4) hours. As an antifermentative and antiseptic measure No. 4 will certainly answer. The following is also suggested as a succedanæum:

8. R. Hydrarggri bichloride, gr. 1-4.

Aquæ destillatæ, f. ℥ iv.

M. S. One (1) teaspoonful every four (4) hours. Also:

9. R. Sodii salicylatis, ʒ i.

Aquæ calidœ, f. ℥ xvi.

M. S. Inject in the bowels twice a day. Of course these are mere suggestions and notes from practice, and as Jacobi justly remarks, "no written rule ever supplies or substitutes brains." With respect to excessive heat and atmospheric vitiation it cannot be disputed that a change of air is of the utmost importance. Not a thousand miles from Atlanta there is a town of no mean import as a summer resort much frequented by mothers and their progeny. There lives within the precincts of that town a physician of considerable ability and reputation. The name of the good doctor is blessed in many a household, and very justly. But I will say *sub rosa* (?) that the change of air has been and is a powerful adjuvant in towering up the reputation of that Babies' Doctor! I will now close this paper by endorsing and recommending Jacobi's food as the only one that has ever given me any satisfaction. It is prepared as follows: Boil a teaspoonful of powdered barley (grind it in a coffee grinder) and a gill of water, with a little salt, for fifteen minutes, strain it, and mix it with half as much boiled milk and a lump of white sugar. Give it lukewarm, through a nursing bottle.

Keep bottle and mouthpiece in a bowl of water when not in use. Babies of five or six months, half barley-water and half boiled milk, with salt and white sugar.

Older babies, more milk in proportion.

When babies are very costive, use oatmeal instead of barley. Cook and strain.

